# Neuromyelitis Optica in a Healthy Female After Severe Acute Respiratory Syndrome Coronavirus 2 mRNA-1273 Vaccine

**DOI:** 10.7759/cureus.17961

**Published:** 2021-09-14

**Authors:** Priscilla Fujikawa, Farhan A Shah, Michalla Braford, Kashyap Patel, Jason Madey

**Affiliations:** 1 Internal Medicine, Lewis Gale Medical Center, Salem, USA; 2 Internal Medicine, Edward Via College of Osteopathic Medicine, Blacksburg, USA; 3 Neurology, Lewis Gale Medical Center, Salem, USA

**Keywords:** nmosd, covid, sars-cov-2 mrna-1273, vaccine, autoimmune

## Abstract

Neuromyelitis optica spectrum disorder is an autoimmune demyelinating disease with high relative prevalence in the East Asian population. Clinical manifestations include optic neuritis, longitudinally extensive transverse myelitis, area postrema syndrome, brainstem syndromes, and diencephalic syndromes.

In this case report, we present a case of neuromyelitis optica spectrum disorder that developed 10 days after the first dose of the severe acute respiratory syndrome coronavirus 2 mRNA-1273 vaccine. The patient was a previously healthy White female, completely independent and functional at baseline. She presented with bilateral lower-extremity numbness/tingling, weakness, and urinary retention. Although her neuromyelitis optica IgG was negative, the MRI was consistent with neuromyelitis optica involving and spanning longitudinally the C6-T2 vertebrae. She was treated with IV steroids and her symptoms improved.

Given the novelty of the COVID-19 vaccines and the paucity of literature regarding their adverse effects, case reports such as ours provide unique information that aids healthcare providers in accurately diagnosing and treating patients, ultimately minimizing long-term neurologic deficits.

## Introduction

Neuromyelitis optica spectrum disorder (NMOSD) is an autoimmune process characterized by severe demyelination affecting the optic nerve and spinal cord [1]. Longitudinally extensive transverse myelitis (LETM) is its most specific presentation, which includes inflammation of the gray matter over three or more contiguous vertebral bodies [2]. We present the case of a 46-year-old female, healthy at baseline, who developed bilateral lower-extremity weakness and urinary retention following the first dose of the severe acute respiratory syndrome coronavirus 2 (SARS-CoV-2) mRNA-1273 vaccine. Her presentation was consistent with NMOSD and thought to be triggered by the vaccine.

## Case presentation

The patient was a 46-year-old White female with a past medical history of vitamin B12 deficiency (on replacement therapy). At baseline, she was able to perform activities of daily living independently and worked as a teacher. Two days after receiving the first dose of the SARS-CoV-2 mRNA-1273 vaccine, she developed constant, shooting, 10 out of 10 upper back pain in between her shoulder blades that radiated to her arms and lower chest. She went to emergency department (ED) and cardiac etiologies of her pain were ruled out with an EKG, troponin, and CT angiogram of chest, abdomen, and pelvis. Three days later, she returned to the ED with paresthesia distal to the T10 dermatome and bilateral upper- and lower-extremity weakness in addition to the previous symptoms. She was given prednisone and a muscle relaxant. The next day, she returned to the ED with persistent symptoms and development of partial urinary retention. She denied any history of trauma, tick bites, or travel.

Her vital signs were normal. On physical examination, abnormal findings included proximal and distal left lower-extremity strength ⅗ and right lower-extremity strength ⅘. There was no weakness in the upper extremities. She had decreased sensation to light touch and sharp materials anteriorly from her feet to the T4 vertebra. Sensation to light touch and sharp materials was also diminished posteriorly from the feet to the T10 vertebra on the left and to the T6 vertebra on the right. There was hyporeflexia of the bilateral ankle, patella, and biceps reflexes. She had poor static and dynamic balance requiring two-people assistance.

**Figure 1:**
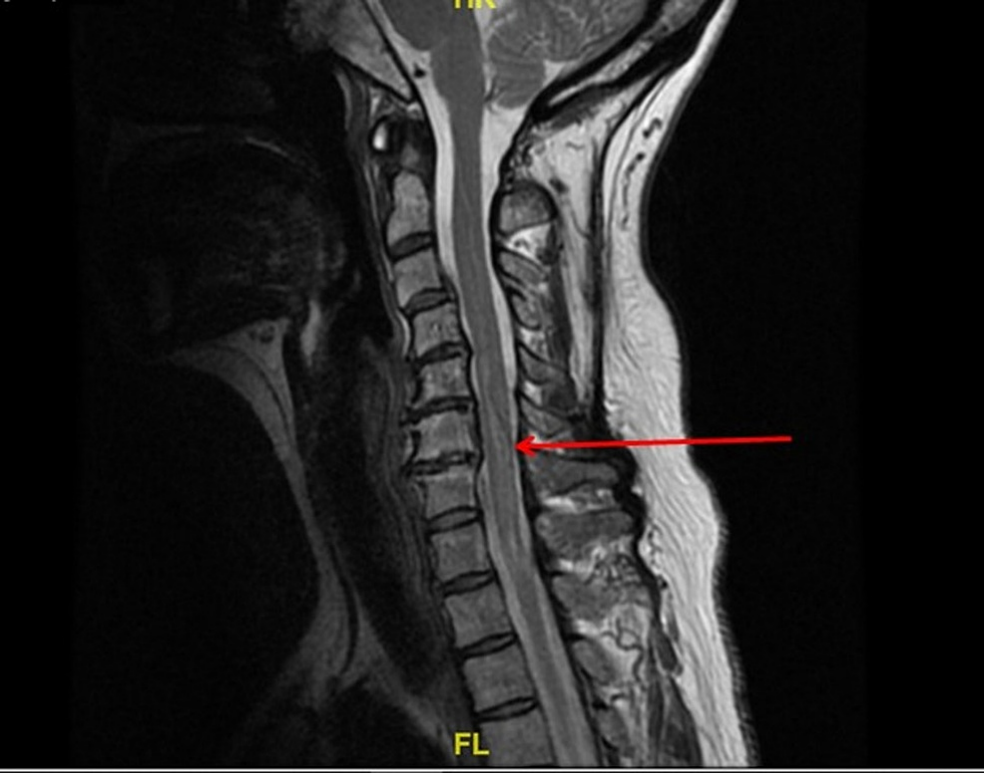
Sagittal, T2 MRI of the cervical spine. Red arrow indicates increased signal within the central gray matter across C6-T2.

Initial labs revealed low vitamin B12 level at 245 pg/mL (reference of range 254-1320 pg/mL). Urinalysis revealed pyuria without bacteria and culture showed at least three organisms, consistent with contamination. Other labs including complete blood count, comprehensive metabolic panel, phosphorus, magnesium, creatine kinase, C-reactive protein, thyroid-stimulating hormone, hemoglobin A1C, aldolase, methylmalonic acid, antinuclear antibody, Jo-1, SS-A/Ro, SS-B/La, ribonucleoprotein, scleroderma, double-stranded DNA, anti-ribosomal, chromatin, centromere B antibodies, complements C3, C4, and neuromyelitis optic IgG (also known as aquaporin-4 [AQP4] antibody) were within normal limits. Cerebrospinal fluid analysis was within normal limits, including a negative venereal disease research laboratory test and undetectable IgG and IgM against *Borrelia burgdorferi*. Brain, lumbar, and thoracic spine MRI were normal without acute pathology. However, T2-weighted MRI of the cervical spine revealed increased, nonexpansile intramedullary signal involving the central gray matter at C6-T2 without enhancement, consistent with NMOSD (Figure 1).

In the interim, the patient was treated with vitamin B12 replacement and, on day 3 of admission, started on methylprednisolone 1 g IV daily. Her proximal and distal strength increased to 4/5 in the lower extremities by day 6 of admission. However, urinary retention and decreased sensation persisted posteriorly up to the T4 vertebra. The patient was ultimately discharged to inpatient rehabilitation on a prednisone taper. She was seen outpatient a month later and her bilateral lower-extremity muscle weakness had resolved.

## Discussion

The prevalence of NMOSD ranges from approximately 0.5 to 4/100,000, and can be up to 10/100,000 in particular racial groups. East Asian populations have a higher prevalence of NMOSD (approximately 3.5/100,000) compared to the White and other Asian populations. The Japanese and Chinese populations share the same human leukocyte antigen (HLA) risk genes for NMOSD: HLA-DPB1*05:01 and HLA-DRB1*16:02. The Black population has a prevalence of up to 10/100,000 compared to 1/100,000 in the White population. The annual incidence of NMOSD among the White population is reported to be approximately 0.5-0.8/million. We did not find population-based studies of NMOSD from Africa in our review. Clinical features of NMOSD are affected by onset age and racial differences. For example, 61% of patients with NMOSD onset <30 years of age present with optic neuritis, while 66% of patients with onset >50 years present with myelitis [3].

Cases of post-vaccination NMOSD similar to ours have previously been reported. These include both de novo attacks and relapses. The association has been observed with the influenza, tetanus/diphtheria, pneumococcal, hepatitis, human papilloma virus, yellow fever, and Japanese encephalitis vaccines. De novo cases are more commonly seen with AQP4 antibody-negative patients while 75% of individuals who experience relapses are seropositive for the AQP4 antibody, and are mainly White females. Onset of relapse symptoms can occur in up to 90 days following vaccination but the incidence is highest at 30 days. Those on preventive immunotherapy for NMOSD have an 81% lower risk of disease relapse following vaccinations [4,5].

NMOSD is a disease characterized by severe demyelination affecting the optic nerve and spinal cord. This process is facilitated by antibodies that attack aquaporin water channels and lead to complement-mediated destruction and subsequent demyelination. AQP4 is the most common aquaporin channel in the brain of mammals [1]. The areas of the central nervous system that are most commonly affected by NMOSD are those where AQP4 channels are abundantly expressed. These regions and their correlated pathologies include the optic nerve (optic neuritis), spinal cord (LETM), dorsal medulla (area postrema syndrome), brainstem (acute brainstem syndromes), and the thalamus/hypothalamus (acute diencephalic syndromes such as narcolepsy) [2]. Optic neuritis is the most common initial symptom, worsened by movement, associated with pain and decreased visual acuity. Optic nerve involvement can be unilateral or bilateral. Approximately 50% of NMOSD patients lose functional vision within five years of disease onset if untreated [6]. NMOSD attacks are very often severe and reach nadir in less than a week. Based on the affected region, symptoms of NMOSD can include intense itching, paresthesia, dysesthesia, pain, seizures, headaches, bladder dysfunction, hyperthermia, galactorrhea, depression, suicidal ideation, and tonic spasms consisting of brief, recurrent and painful episodes of increased muscle tone with abnormal posturing of the affected limb. LETM is the most specific form of presentation of NMOSD, which includes inflammation affecting the central gray matter, extending over three or more contiguous vertebral bodies [2,6].

Diagnostic criterion for NMOSD is based on AQP4-IgG status. In the presence of AQP4 antibodies (AQP4-Ab), one of six core clinical criteria is required to diagnose NMOSD: optic neuritis, acute myelitis, area postrema syndrome, brainstem syndrome, symptomatic narcolepsy or acute diencephalic syndrome with NMOSD lesions on MRI, symptomatic cerebral syndrome with NMOSD lesions [2]. In the absence of AQP4-Ab or unknown serology, the requirements are two core clinical criteria including optic neuritis, acute myelitis with LETM, or area postrema syndrome and dissemination in space and MRI findings consistent with NMOSD [7].

MRI findings are typically localized in regions of the brain with high expressions of AQP4. The most common findings are seen on T2-weighted or fluid-attenuated inversion recovery sequences. Nonspecific, small, hyperintense dots and patches appear in the subcortical and deep white matter. Additional findings include diencephalic lesions that are located around the third ventricle and cerebral aqueduct, dorsal brainstem lesions across from the fourth ventricle, periependymal lesions surrounding the lateral ventricle, hemispheric white matter lesions, lesions involving the corticospinal tract, and enhancing lesions often described as “cloud-like” enhancements. Optic nerve findings vary from nonspecific optic nerve sheath thickening to hyperintensities but are not diagnostic of NMOSD. Hyperintensity on T2-weighted sequences and hypointensity on T1-weighted sequences are found frequently in the cervical and upper thoracic spinal regions compared to lower thoracic and lumbar regions. One of the most diagnostic findings on MRI is the presence of LETM spanning three or more contiguous vertebral segments. The area affected is predominantly gray matter, attributed to the abundance of AQP4 in the gray matter and glial cells that are associated with the ependymal cells of the central canal [8].

The goals of treatment are to suppress inflammation, minimize central nervous system damage, and improve long-term neurological function. The first-line therapeutic agent is corticosteroids: methylprednisolone IV 1,000 mg for five days followed by an oral steroid taper for 2-8 weeks [9]. If steroids are not effective, it is reasonable to transition to plasma exchange therapy. Its use following high-dose steroids is more effective than steroids alone in achieving baseline neurological function. Improvement is expected with a reduction in inflammation and edema leading to gradual neurological improvements over the following six to 24 months [9].

Traditional secondary prevention includes immunosuppressant therapy, such as azathioprine or mycophenolate mofetil. However, recent research shows that rituximab and tocilizumab are superior in relapses and disease progression, which has created a trend away from azathioprine [10]. Eculizumab was recently approved for the treatment of NMOSD. This was based on the Prevention of Relapses in Neuromyelitis Optica (PREVENT) trial where it reduced the risk of relapses in AQP4-IgG seropositive adults compared to placebo. Additional therapies approved in 2020 include satralizumab and inebilizumab. Suggested sequencing includes eculizumab followed by satrilizumab for long-term immunomodulation with reservation of inebilizumab for breakthrough disease. If severe enough, autologous hematopoietic stem cell bone marrow transplantation can be considered [11,12].

Considering the temporal association between administration of the vaccine, onset of patient’s symptoms, and previous reports of post-vaccination NMOSD, we have strong evidence to conclude that this patient’s NMOSD was triggered by the SARS-CoV-2 mRNA-1273 vaccine. Not only did she have multiple symptoms consistent with this disorder, including pain, paresthesia, weakness, and urinary retention, but she also had the most specific form of its presentation: LETM. Her negative AQP4-Ab serology made her diagnosis more challenging as she only had one core clinical criterion. However, her MRI results of hyperintensity spanning the gray matter of C6-T2 are the most specific finding of NMOSD, the extensive work-up performed excluded alternative diagnosis, and her symptoms improved after initiation of corticosteroids. Therefore, we are confident that this was a case of post-vaccination NMOSD. Immunosuppressants were not initiated during this acute attack as the discussion about secondary prevention was more appropriate in the outpatient setting.

## Conclusions

This case report provides crucial information on the possible adverse effects associated with the SARS-CoV-2 mRNA-1273 vaccine. Post-vaccination myelitis and other neurologic reactions are rare. Nonetheless, early recognition is important as treatment can sometimes curtail long-term disability. Considering the novelty of these vaccines, there is a paucity of literature on this topic. NMOSD is a severe and rapidly progressive disease that can result in profound disability, including loss of vision, paralysis, and seizures. Therefore, a high degree of suspicion is key to accurately diagnose and intervene in order to prevent long-term neurological complications.
